# Obstetric interventions in two groups of hospitals in Catalonia: a cross-sectional study

**DOI:** 10.1186/1471-2393-14-143

**Published:** 2014-04-15

**Authors:** Ramón Escuriet, María Pueyo, Herminia Biescas, Cristina Colls, Isabel Espiga, Joanna White, Xavi Espada, Josep Fusté, Vicente Ortún

**Affiliations:** 1 Directorate-General for Health Planning and Research, Ministry of Health of the Government of Catalonia, Pompeu Fabra University, Travessera de les Corts, 131-159, Pavelló Ave Maria, Barcelona, 08028, Spain; 2 Directorate-General for Health Planning and Research, Ministry of Health of the Government of Catalonia, Barcelona, Spain; 3 Catalan Agency for Health Information, Assessment and Quality, Barcelona, Spain; 4 Observatory on Women’s Health, Subdirectorate for Quality and Cohesion, Ministry of Health, Social Services and Equality, Madrid, Spain; 5 Centre for Research in Anthropology (CRIA-IUL), Lisbon, Portugal; 6 Visiting Fellow, King’s College London, London, UK; 7 Fundació Hospital Asil de Granollers, Granollers, Spain; 8 Faculty of Economic and Business Sciences, Pompeu Fabra University, Barcelona, Spain

**Keywords:** Obstetric intervention, Caesarean section, Variability, Hospital birth

## Abstract

**Background:**

Childbirth assistance in highly technological settings and existing variability in the interventions performed are cause for concern. In recent years, numerous recommendations have been made concerning the importance of the physiological process during birth. In Spain and Catalonia, work has been carried out to implement evidence-based practices for childbirth and to reduce unnecessary interventions.

To identify obstetric intervention rates among all births, determine whether there are differences in interventions among full-term single births taking place in different hospitals according to type of funding and volume of births attended to, and to ascertain whether there is an association between caesarean section or instrumental birth rates and type of funding, the volume of births attended to and women’s age.

**Methods:**

Cross-sectional study, taking the hospital as the unit of analysis, obstetric interventions as dependent variables, and type of funding, volume of births attended to and maternal age as explanatory variables. The analysis was performed in three phases considering all births reported in the MBDS Catalonia 2011 (7,8570 births), full-term single births and births coded as normal.

**Results:**

The overall caesarean section rate in Catalonia is 27.55% (CI 27.23 to 27.86). There is a significant difference in caesarean section rates between public and private hospitals in all strata. Both public and private hospitals with a lower volume of births have higher obstetric intervention rates than other hospitals (49.43%, CI 48.04 to 50.81).

**Conclusions:**

In hospitals in Catalonia, both the type of funding and volume of births attended to have a significant effect on the incidence of caesarean section, and type of funding is associated with the use of instruments during delivery.

## Background

Provision of childbirth care is an important element of hospital activity. Quality and safety criteria have gradually been applied to birth, together with increasingly more technological resources. The current childbirth care model tends to consider normal birth in retrospect, i.e. a birth is regarded as normal once it is over and there has been no problem. This approach has contributed to the transformation of a physiological event into a medical and surgical process [1]. Support to women (both those with or without obstetric risk), is provided in a highly technological environment [2] and care is organised and applied systematically to the mother and the newborn. In the context of a normal labour, this mode of care could interfere with the physiological process and introduce risks arising from unnecessary interventions [3]. Moreover, there is growing concern about the existing variation in childbirth care practice and the possible costs, in both health and economic terms, of following an interventionist model when attending to women without any obstetric risk in a highly technological environment [4-9].

In recent years, scientific-based recommendations have emerged which promote a childbirth care model that respects the physiological process. In some countries these recommendations have been progressively and variously incorporated into health policies and documents of different professional organisations. Also policy makers have taken into consideration the demands made by many women’s associations to de-medicalize childbirth. Access to information varies in each setting, but currently there is a broad dissemination of information related to best practice for normal childbirth care in most industrialised countries, including documents intended to help women take informed decisions about how they want to be treated during delivery [10-13].

The definition of “normal birth” varies depending on the context. Inclusion and exclusion criteria to consider when a labour and birth is “normal” may differ, making it difficult to study the process of normal birth care and outcomes. Most agreed definitions include women whose labour starts spontaneously, progresses spontaneously without drugs, and who give birth spontaneously [14]. In Spain a similar definition, with special additional mention of “respectful birth care”, has been agreed by midwives associations and by obstetricians societies who have incorporated the concept of normality during pregnancy in their consensus definition [10,15].

In 2007, the *Strategy for Assistance at Normal Childbirth in the National Health System* (SANC) [16] was published in Spain, with a series of recommendations aimed at improving the support given to women during delivery and birth. While the SANC does not include an explicit definition of normal birth, t the strategy is founded on the principle that childbirth is a normal process that should only be intervened in when necessary. This guiding principle has enabled the transformation of the existing childbirth assistance model to one which focuses more on mothers’ needs, and efforts have been made to involve women in decision-making about the type of assistance they want. Since its inception the SANC has promoted the training of professionals and the strategic adaptation of hospitals as measures to facilitate good practice in childbirth care. In Catalonia, a refocusing of maternity services towards a less interventionist approach was initiated in 2007 [17]. In line with SANC, this shift resulted in the progressive implementation of a more women-focused model predicated upon the minimal necessary intervention during delivery. This approach became the project for normal childbirth care, gradually introduced across the region. Currently, there are 44 hospitals in the public health network in Catalonia with maternity healthcare services. This public health network includes 6 publicly owned hospitals and also 38 state-assisted hospitals which provide public and private health services. The Ministry of Health of the Government of Catalonia provided financial support to 32 of these hospitals to improve their facilities and to adapt maternity wards where women labour and deliver in line with the normal birth approach.

Since its conception, this project has been monitored and coordinated by the Ministry of Health of the Government of Catalonia, a process requiring constant contact with the leaders of each health region, service providers and maternal health professionals responsible for childbirth assistance. This follow-up identified the need to develop appropriate indicators for evaluation, to update recommendations on best practice on a regular basis and to promote the dissemination of these recommendations among professionals and the wider population.

Work is currently ongoing in Catalonia to update the clinical practice guideline for normal childbirth assistance, disseminate updated recommendations, and conduct monitoring visits to the 32 hospitals that received economic support. Hospital discharge data from 2011 have also been explored and part of this analysis is presented in this current study. Furthermore, in 2010 the Ministry of Health, Social Services and Equality performed an internal evaluation of the implementation of the SANC in Spain overall. This assessment identified some improvements, but also highlighted the need for further work to be done in order to reduce the rates of certain obstetric interventions, which remain above recommended levels. Concrete recommendations for improvement include:

•Protocols must be updated periodically.

•All interventions during labour and birth should be recorded.

•Overall prevalence of instrumental births should decrease and vacuum should be used rather than forceps.

•The existing recommendation that episiotomy should not be used routinely needs to be reinforced.

•Overall C-section rates should decrease in most of the hospitals.

This countrywide evaluation provides useful context for the ongoing evaluation of childbirth care in Catalonia. For this purpose, and as far as SANC and the project for normal childbirth care in Catalonia are focused on model of care but do not provide a definition on “normal birth”, in our study we have considered the same criteria as used in Minimum Basic Data Set (MBDS) for normal birth, when a woman with a straightforward pregnancy delivers at term, between 37 to 40 weeks of pregnancy, vaginally and without instrumentation a single newborn in cephalic presentation.

The MBDS includes information from all forty-four public hospitals and twenty private hospitals (out of 27), representing 98.9% of all deliveries in Catalonia (MBDS 2011: 78,570 registered; Catalan Statistics Institute 2011: 79,413 registered births). Funding of both public-owned and state-assisted hospitals is based on activity and paid by the Catalan Health Service according to the number of discharges. Regarding private hospitals, women are responsible for all costs of childbirth care received either, most commonly, by purchasing private health insurance, or through direct payment to the company (or health care professional) providing the service. Private hospital charges can vary but are largely based on interventions and length of stay.

### Objectives

To identify obstetric intervention rates (C-section, use of instruments for delivery and performance of episiotomy) in women giving birth in hospitals in Catalonia in the course of 2011.

To explore whether there were significant differences in intervention rates in single births between 37-42 weeks between hospitals according to type of funding, volume of births attended to and women’s age.

## Methods

Retrospective observational cross-sectional study of all births in 2011 identified in the hospital discharge register in Catalonia, the MBDS, a register of all acute care hospital discharges. The register is mandatory for all public hospitals and provides the basis for reimbursement. Each hospital discharge is registered with administrative information on the patient, hospital episode and the hospital identification. The diagnoses are coded according to the *International Classification Diseases, Ninth Revision, Clinical Modification* (ICD-9-CM edition).

Given the need to measure available and comparable indicators when analysing “normal birth” we had to adopt the available reported code in Minimum Basic Data Set which best approximates to this concept, assuming its limitations, but in the assurance that when this code is used, no obstetric risk has been identified during pregnancy and birth.

The unit of analysis considered in this study was the hospital. For the analysis, hospitals were classified into two groups according to funding (public or private), and four strata were defined for each group according to the volume of births attended to *(stratum 1* (S1)*:* ≤ 600 births; *stratum* 2(S2): > 600 to ≤ 1200 births; *stratum* 3(S3): > 1200 to ≤ 2400 births; *stratum* 4(S4) > 2400 births). Strata were defined following the same criteria used in the SANC. During data extraction a total of 1,444 births in public hospitals which were not publicly funded was identified. These were explicable due to the coexistence of private activity in some public hospitals, but were excluded from the analysis. A total if 252 births were also excluded due to inconsistent reporting.

To elaborate the indicators, the codes of the revised ICD-9-CM edition, which were used for the MBDS in 2011, were grouped together. The grouping of codes makes it possible to identify the dependent variables that correspond to the various obstetric interventions analysed (C-section, forceps, vacuum, unspecified instrument and episiotomy) and normal birth. Type of hospital funding, stratum according to volume of births and the mother's age were taken as independent or explanatory variables.

**Figure 1:**
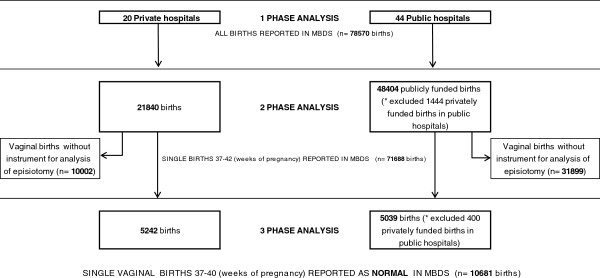
Population distribution.

A three-phase analysis was performed to analyse hospital performance in terms of obstetric intervention, Figure 1. The first phase yielded the result of the indicators out of total births in Catalonia, the second phase gave the result of the indicators out of all births with a single newborn between 37-42 weeks of gestation, and the final phase gave the result of the indicators out of single births between 37-40 weeks of gestation coded as normal. The consideration of normal birth in the MBDS registry is defined as spontaneous onset of labour, cephalic presentation of the foetus and vaginal birth of a single live newborn between 37-40 weeks of gestation without obstetric risk [18]. This coding limitation on duration of pregnancy comes from the specific, standard coding proceedings utilised by the Catalan Health Service, and excludes deliveries beyond 40 weeks that could also be coded as normal.

The episiotomy intervention analysis was performed on non-instrumented vaginal births and normal births.

### Groupings of diagnoses and main procedures

**Caesarean section:** (74.0-74.2, 74.4, 74.99)

**Forceps:** (72.0-72.6, 73.3)

**Vacuum:** (72.7)

**Unspecified instrument:** (72.8-72.9)

**Episiotomy:** (73.6)

**Normal birth:** 650, accompanied by secondary diagnosis **single live birth**: V27.0

### Details of ethical approval

This study was exempt from Ministry of Health of the Government of Catalonia Ethics Committee review as it used publicly available, anonymised data.

### Analysis

A descriptive analysis of the obstetric interventions was performed in each group and for each stratum, giving the mean and the confidence interval (95%) of each dependent variable.

A bivariate analysis was performed to obtain the probability of intervention. The explanatory variables considered were: type of funding, the hospital stratum attending the birth and the women's age. Caesarean section and the use of an instrument for delivery were regarded as dependent variables. This last variable grouped the use of forceps, vacuum and unspecified instrument together. The relationships between categorical variables were analysed using the chi-square method and between quantitative variables by the Student’s t test.

A logistic regression analysis was performed on the population of full-term single births to determine the association between caesarean section and the use of instruments with maternal age, type of funding and birth volume of the hospital where the delivery occurred. The odds ratio and the confidence interval were calculated for each variable.

When exploring episiotomies, we calculated the variation rate, ratio between the highest and the lowest observed average in strata for each group of hospitals.

PASW Statistic 18 statistical package was used for data analysis.

## Results

The first phase identified a total of 78,570 births recorded in Catalonia in 2011: 24,155 in private hospitals and 54,415 in public hospitals.

The distribution of total births in Table 1 evinces a significant difference in mean age and mean days of stay between public and private hospitals. This difference is not significant in the distribution of full-term single births or normal single vaginal births analysed across the two types of hospital.

**Table 1:** Birth distribution and characteristics according to type of hospital and type of funding

	**Number of hospitals**		**Total births Catalonia, 2011**				**Single births 37-42 (wp)**				**Normal single vaginal birth 37-40 (wp)**			
	**(N)**		**(N)**		**Average maternal age (years) (CI)**	**Average length of stay (days) (CI)**	**(N)**		**Average maternal age (years) (CI)**	**Average length of stay (days) (CI)**	**(N)**		**Average maternal age (years) (CI)**	**Average length of stay (days) (CI)**
Public hospitals														
	All births reported in MBDS						Publicly funded births in public hospitals							
Stratum S1	12	44	2814	54415	30.75 (30.71-30.80)	3.05 (3.04-3.07)	2365	48404	30.6 (18.9-42.4	2.85 (0.56	532	5039	28.9 (18.8-39.7	2.40 (0.95-3.85
Stratum S2	14		9145				8124				1485			
Stratum S3	17		26385				23790				2315			
Stratum S4	5		16071				14125				707			
Private hospitals														
	All births reported in MBDS						Private funded births in private hospitals							
Stratum S1	9	20	2205	24155	33.68 (33.63-33.74)	3.56 (3.53-3.58)	1931	21840	33.6 (25.7-41.4)	3.43 (0.98-5.88)	703	5242	32.8 (25.5-40.1)	2.74 (1.33-4.15)
Stratum S2	3		2294				2127				387			
Stratum S3	2		3217				2988				479			
Stratum S4	6		16439				14794				3673			

The table shows a greater number of full-term single births coded as normal births among births in private hospitals. The analysis of this distribution gives a rate of 24.0% (CI 22.89-25.12) of normal full-term single births among births in private hospitals and a rate of 10.41% (CI 9.57-11.25) among births in public hospitals This difference in distribution was found for all strata according to volume of activity with the exception of stratum 2. This phenomenon could be explained because public hospitals are particularly well prepared to attend women who present high obstetric risk and complex pathologies, and are receiving most of these women In Catalonia approximately 30% of all births occur in private hospitals every year.

With regard to the obstetric interventions analysed, Table 2 presents the rate of each intervention with their respective confidence intervals out of overall births in the MBDS in each stratum of the volume of births attended to. C-section and the use of vacuum were more frequent among hospitals with the lowest volume of activity. An examination of the rates of interventions that are mutually exclusive (C-section, forceps, vacuum and unspecified instrument), illustrates the level of intervention in each stratum, and reveals that in hospitals with a lower volume of activity (S1) some kind of surgical or instrumental intervention occurred in almost half of births.

**Table 2:** All births

		**Number of hospitals**		**Number of births**		**Caesarean section**				**Forceps**				**Vacuum**				**Unspecified instrument**			
		**(N)**		**(N)**		**(N)**		**Rate (CI)**		**(N)**		**Rate (CI)**		**(N)**		**Rate (CI)**		**(N)**		**Rate (CI)**	
Operative and instrumental births	All hospitals																				
		All births reported in MBDS																			
	Stratum S1	21	64	5019	78570	1745	21643	34.77% (33.45-36.09)	27.55% (27.23-27.86)	203	5284	4.04% (3.50-4.59)	6.73% (6.55-6.90)	364	3519	7.25% (6.53-7.97)	4.48% (4.33-4.62)	169	3449	3.37% (2.87-3.87)	4.39% (4.25-4.53)
	Stratum S2	13		11439		2756		24.09% (23.31-24.88)		493		4.31% (3.94-4.68)		398		3.48% (3.14-3.82)		593		5.18% (4.78-5.59)	
	Stratum S3	19		29602		7049		23.81% (23.33-24.30)		2381		8.04% (7.73-8.35)		1232		4.16% (3.93-4.39)		1447		4.89% (4.64-5.13)	
	Stratum S4	11		32510		10093		31.05% (30.54-31.55)		2207		6.79% (6.52-7.06)		1525		4.69% (4.46-4.92)		1240		3.81% (3.61-4.02)	

Obstetric intervention rates by stratum.

**Figure 2:**
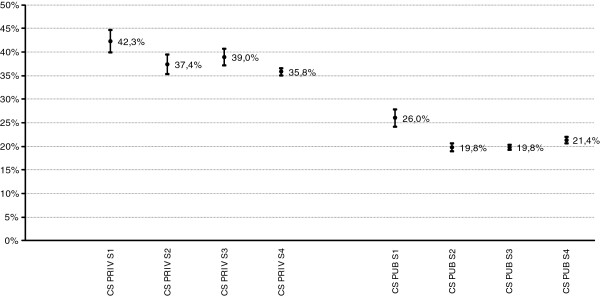
**Single birth 37-42 weeks of pregnancy (wp).** Volume-stratum C-section rates. Public and private hospitals.

Figure 2 includes caesarean section rates for disaggregated full-term single births in each hospital group according to the volume of births in each stratum. Both hospital groups present the highest caesarean rates in stratum S1, which is significantly different from other strata within each group. Private hospitals have substantially higher rates of caesarean section across all strata.

When analysing instrumental birth rates amongst 37-42 weeks of pregnancy (wp) single vaginal births we found a significant variation in instrumental birth rates in both groups of hospitals, but the overall range of instrumental vaginal births is narrow. Higher levels of vacuum deliveries can be observed in stratum S1 (15.6%) and stratum S2 (15.1%) in the private hospitals group.

**Figure 3:**
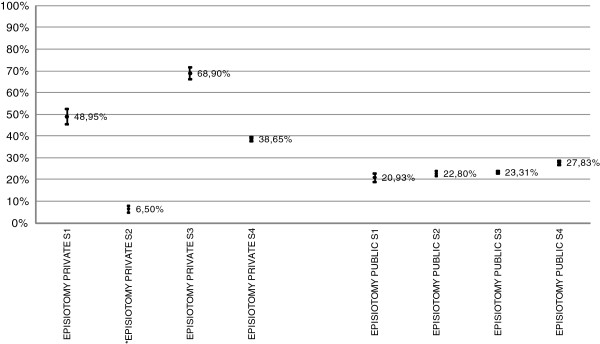
**Single vaginal birth without instrument 37-42 weeks of pregnancy (wp).** Volume-stratum episiotomy rates. Public and private hospitals.

As can be seen in Figure 3, the episiotomy rates reported in public hospitals are higher the greater the volume of births attended to, although they always range from 20.93% to 27.83%, giving a variation rate of 0.75. With regard to episiotomy rates reported in private hospitals, the graph shows a wide range, from 6.50% to 68.9%, giving a variation rate of 10.6. Variation rate is a very simple data form but very intuitive and this result suggests that episiotomy may not be well reported in some private hospitals, and reinforces the existing government recommendation that all interventions should be properly recorded.

Table 3 shows the episiotomy rate amongst full-term single vaginal births that have been recorded as completely normal. The data are presented in aggregate form for each hospital group. The table shows a higher rate of episiotomy among deliveries in private hospitals, suggesting that the recommendation for not conducting episiotomy routinely has not been adopted by private hospitals. This is particularly important given, as noted earlier, that private hospitals currently attend a higher proportion of normal births (Table 4).

**Table 3:** Normal birth 37-40 weeks of pregnancy (wp)

	**Number of hospitals**	**Registered normal births**	**Episiotomies**		
	**(N)**	**(N)**	**(N)**	**Rate**	**CI**
Public hospitals					
Normal single vaginal birth 37-40 (wp)	Publicly funded births in public hospitals				
	44	5039	1442	28.62%	27.28-29.95
Private hospitals					
Normal single vaginal birth 37-40 (wp)	Private funded births in private hospitals				
	20	5242	2003	38.21%	36.78-39.64

Episiotomy rate.

**Table 4:** Single birth 37-42 weeks of pregnancy (wp)

	**Maternal age**			**Type of financing**			**Volume-stratum**		
	**OR**	**CI (95%)**	**p value**	**OR**	**CI (95%)**	**p value**	**OR**	**CI (95%)**	**p value**
Caesarean section	1.043	1.039-1.047	0.000	2,014	1.940-2.090	0.000	0.976	0.957-0.995	0.015
Instrumental vaginal birth	0.98	0.976-0.984	0.000	1,442	1.377-1.510	0.000	1.016	0.992-1.040	0.198

C-section and instrumental vaginal birth association with maternal age, type of financing and volume-stratum.

The logistical regression analysis indicates an association between the caesarean section dependent variable and the independent variables considered. This association shows that the likelihood of having a caesarean section decreases as the volume of births attended to in the hospital increases, OR 0.976 (95% CI 0.957-0.995). In contrast, the likelihood of caesarean section increases with increasing maternal age, OR 1.043 (95% CI 1.039-1.047) and private hospitals OR 2.014 (CI 1.940-2.090).

As for the instrumental birth variable, increased maternal age reduces the probability of having an instrumental birth overall, OR 0.98 (95% CI 0.976-0.984), whereas in private hospitals this probability increases, OR 1.442 (95% CI 1.377-1.510). The relationship between the probability of having an instrumental birth and the volume of deliveries in the hospital is not clear.

## Discussion

### Clinical practices

Our study was conceived with the aim of defining indicators that permit a periodic assessment of normal childbirth in hospitals in Catalonia using the data systematically recorded in the MBDS. Parallel to the study, follow-up work was undertaken to analyse the situation in the 32 hospitals of Catalonia currently involved in the project for normal birth care. These follow-up visits are intended to collect information about the available infrastructure and resources, organisation of services and care and the nature of current activities. This work identified variations in the way certain procedures, such as amniotomy and medical induction of birth are recorded as well as the coding of “normal birth”. It was observed, for example, that some healthcare professionals consider induction of birth and amniotomy as minor interventions, while in other cases there was a tendency not to use the code of normal birth, even if it fitted with the event, in other words when no intervention has been performed, which suggests there may be under-reporting of normal birth cases.

Data analysed in this study show how the prevalence of episiotomy and C-section are far removed from the standards agreed to at the SANC, which advocates a maximum rate of 15% in both cases. For this reason, these may act as important indicators of the successful implementation of best practice for normal childbirth.

Variations in the performance of caesarean sections and the factors influencing this disparity have become a major area of study in recent years. Research in Spain in 2009 showed that the increase in C-sections is due both to a rise in the number of elective primary caesarean sections as well as repeat caesarean sections. However, while in public hospitals the increased incidence of caesarean sections is proportionately lower than the overall increase in number of births, in private hospital settings the increase in C-sections far outweighs the total number of births [19]. This reported difference in the incidence of C-sections according to public or private hospital care is confirmed by our analysis, which identified the highest rates of caesareans occurring in private hospitals. This finding tallies with the results of various earlier comparative studies and suggests that factors such the profile of women who seek private care, the budgeting of private services and health professionals clinical experience may be relevant in elucidating the differences identified [20]. It is also striking to note, however, that private hospitals have both the highest rates of C-section and of normal births. This finding suggests the need for further research into the range of factors influencing care provision in private settings.

Other factors associated with the organisation of services that might cause intra-hospital variations also need to be taken into account [21]. Particular characteristics of hospitals with a low volume of activity, such as health professionals being on call, limited resources and problems in transferring problematic cases to larger hospitals due to their geographical situation may influence the increased C-section rate found in this stratum across both groups and should be taken into account when discussing how low-volume hospital maternity services are organised in Catalonia.

On the other hand the prevalence of obstetric interventions may be influenced by the use of certain procedures during birth, particularly in healthy, low-risk women, which trigger a cascade of subsequent interventions. The use of epidural analgesia or medical induction and stimulation, for example, may increase rates of instrumental birth with episiotomy and caesarean, also resulting in a rise in the cost of assistance [22-24]. The decision to use certain procedures may, in turn, be influenced by service providers’ model of management based on specific planning criteria and the previous training of health professionals [25-27].

As for the type of instrumentation, this study found a greater use of vacuum in private settings, whereas in public hospitals forceps were used more frequently. This variation might not be due to clinical indications since, as stated in the results, we found that private funding increases the likelihood of having an instrumental birth per se. While the increase in maternal age appears to reduce the probability of instrumentation during birth, this could also be explained by the fact that an increase in age entails a greater likelihood of C-section, hence as maternal age increases the performance of a caesarean section may be prioritised.

Other studies have also identified further factors influencing intervention levels besides the funding characteristic of hospitals, such as the profile of the professional/s caring for low-risk women or the particular unit where the women give birth [28,29].

The distribution of the episiotomy rate found among vaginal full-term single births presents a different pattern between the two hospital groups studied. Episiotomy rates in public hospitals show a variation rate well below that demonstrated by private hospitals. This is difficult to explain. The lower rates observed in public hospitals may be due to the progressive introduction of the non-routine use of episiotomy recommendation, as detailed in the recent SANC evaluation, which reported a rate of 41.9% in vaginal, non-instrumental births of between 37 to 42 weeks in 2009. This reduction was also identified in the latest Euro-Peristat report, [30] which revealed a rate of 43.0% for Spain (data from included regions) in 2010, a significant reduction since 2004. While this current study reveals lower rates of episiotomy for single-term births reported as normal in Catalonia there is evidently ample room for improvement in the implementation of the recommendations for normal childbirth care.

### Maternal age

Maternal age of above 40 years is associated with risk during birth and increases the likelihood of having a caesarean section. However, it cannot be asserted that increased C-section rates are due to a real increase in obstetric complications, as there may be other secondary factors influencing this outcome, which are related to the attitudes of clinicians and women themselves [31-33]. The comparison of C-section rates among different countries confirms this interpretation; the inconsistencies identified suggest that the relationship between the number of mothers aged above 40 and C-section rates may be due to factors other than obstetric risk [34]. Though this study was not designed to specifically study the relation between maternal age and caesarean section, this hypothesis might serve as a basis for further research to explore whether age, by itself, without other risk factors, influences clinical decision-making on delivery method, and if this is the case at what age the tendency towards a greater use of C-section begins or increases, and in what settings it is most prevalent.

### Volume of activity in the hospitals

The hospital as an independent variable underlines the overall effect of the organisation in the likelihood of medical intervention occurring during delivery; [35] there is an assumed bias involved in analysing individual variables and effect variables subject to this “cluster” effect. In this current study, the analysis assumes this bias and therefore hospitals were grouped according to the volume of births attended to, enabling us to compare behaviour across the different strata of each hospital group.

The rates of obstetric intervention in full-term single births found in the settings studied show a higher rate of caesarean sections in hospitals with a lower volume of births, which conforms to the internal assessment of the SANC and other studies conducted in Spain [36]. In terms of instrumental births, higher intervention rates can be seen in the group of privately funded hospitals, and within this group the highest level of intervention was observed among those with fewer births. In view of the spiralling debate on obstetric outcomes in centres with a low volume of activity, it must be remembered that in the Catalonian context there are no “birth centres” or other specific units separate from hospitals which offer maternity care to women of low risk and which typically attend to a limited volume of births. This means that the results concerning the hospitals included in the current study cannot be compared to other types of units with a similar volume of births that are organised differently and have very different outcomes in terms of obstetric intervention [37,28,39].

### Records

As outlined, the variability in obstetric intervention may be affected by factors such as user expectations, unnecessary or inappropriate assistance, the culture of medical practice, the organisation of care provision and available resources. In addition to these issues, current variations in the recording of procedures [35,40-42] render it difficult to accurately evaluate all aspects maternal health services. Even so, most of the data contained in hospital discharge records on the mode of birth can be considered reliable for the purposes of evaluation. However, after comparing the data available from the MBDS to those obtained directly from each hospital service, poor recording of the performance of amniotomy and induction of labour was detected (of the 64 hospitals included, 8 do not record inductions and 31 do not record amniotomies), hence these data were explored but not included in this study.

### Other factors

When attempting to explain variations in the rates of obstetric interventions in full-term single births, the type of funding in the hospital where the assistance is provided should be taken into account, as should the obstetric risks and clinical conditions that may arise during birth. The associations between type of hospital funding, other differential aspects between public and private hospitals, and other factors that may condition obstetric outcomes remain to be studied.

### Limitations

The data analysed are those recorded in the hospital discharge reports. A deficient recording of episiotomies was detected in three of the private hospitals included in stratum S2, which explains the variation rate found in this group for this indicator.

Although the assignment of the corresponding code to the diagnosis of normal birth is assumed to be correct, under-recording of this diagnosis has been identified, together with the bureaucratic limitation that births surpassing 40 weeks of gestation cannot be recorded with this code. It is therefore likely that many of the births not assisted by C-section or instrumentation currently not recorded as normal within the MBDS could be recorded as such. It would be advisable to improve the coding system in order to facilitate consistent monitoring and assessment.

This study did not consider clinical conditions, as the objective was to analyse intervention rates. The standards recommended in the SANC on the different obstetric interventions discussed in this paper are assumed. These standards are useful as a reference to identify high intervention rates.

Parity and previous mode of delivery was not included in the data base and could not be considered for the analysis.

## Conclusions

The volume of births attended to in hospitals and the type of funding of the institution has a significant effect on the incidence of caesarean sections in Catalonia; the type of funding of the hospital also influences the incidence of instrumental births.

### Implications for research

The findings of this study suggest the need for further examination of factors associated with funding and the organisation of childbirth services which are influencing obstetric intervention.

Likely population distribution in the two groups of hospitals indicate the need for further research on factors, such as socio-economic status, that may be influencing women’s choice of public or private childbirth care.

The variables of previous mode of delivery and parity could not be explored because they were not available on our database. Such variables could have an impact in outcomes in terms of obstetric intervention and could be explored in further research.

### Implications for practice

The use of procedures and interventions in maternity service delivery should be reviewed, especially in hospitals that attend to a smaller volume of births.

Given the identification of a tendency to not record certain interventions, the consistent diagnosis and reporting of normal birth as well as procedures such as medical induction of birth and amniotomy should be improved.

## Competing interests

The authors declare that they have no conflicts of interest.

## Authors’ contributions

RE conceived, designed and coordinated the study. MP and RE have been involved in acquisition of data and performed statistical analysis. RE and JW drafted the manuscript. HB, CC, IE and XE revised it critically for important intellectual content. JF and VO revised the manuscript and have given the final approval of the version to be published. All authors read and approved the final manuscript.

## Authors’ information

RE is coordinating the project for normal birth assistance in Catalonia that involves 32 public hospitals, and is also involved in the EU COST Action IS0907 *Childbirth Cultures, Concerns, and Consequences: Creating a dynamic EU framework for optimal maternity care*.

## Pre-publication history

The pre-publication history for this paper can be accessed here:

http://www.biomedcentral.com/1471-2393/14/143/prepub

## References

[B1] Grupo de trabajo de la Guía de Práctica Clínica sobre la atención al parto normal Guia de práctica clínica sobre la atención al parto normal 2010 Vitoria: OSTEBA, AVALIA-T

[B2] Goberna Tricas J Autonomia, heteronomía y vulnerabilidad en el proceso de parto Rev Enferm 2012 6 71 78

[B3] Romano AM Lothian JA Promoting, protecting and supporting normal birth: a look at the evidence J Obstet Gynecol Neonatal Nurs 2008 37 94 104 10.1111/j.1552-6909.2007.00210.x 18226163

[B4] Rossen J Okland I Nilsen OB Eggebø TM Is there an increase of postpartum hemorrhage and is severe hemorrhage associated with more frequent use of interventions? Acta Obstet Gynecol Scand 2010 89 1248 1255 10.3109/00016349.2010.514324 20809871

[B5] Mikolajcyk R Schmedt N Zhang J Lindemann C Langner I Garbe Regional variation in caesarean deliveries in Germany and its causes http://www.biomedcentral.com/1471-2393/13/99 10.1186/1471-2393-13-99 PMC3652783 23634820

[B6] Wagner M Fish can’t see water: the need to humanize birth Int J Gynaecol Obstet 2001 75 Suppl 1 25 37 10.1016/S0020-7292(01)00519-7 29645271

[B7] Kozhimannil KB Law MR Viming BA Cesarean delivery rates vary tenfold among US hospitals: reducing variations may address quality cost issues Health Aff 2013 32 527 535 10.1377/hlthaff.2012.1030 PMC3615450 23459732

[B8] Lutomski JE Morrison JJ Lydon-Rochelle MT Regional variations in obstetrical intervention for hospital birth in the Republic of Ireland, 2005-2009 http://www.biomedcentral.com/1471-2393/12/123 10.1186/1471-2393-12-123 PMC3541199 23126584

[B9] Rossignol M Moutquin JM Bougrassa F Bédard MJ Chaillet N Charest C Ciofani L Pilon MD Gagné GP Gagnon A Gagnon R Senikas V Preventable obstetrical interventions: how many caesarean sections can be prevented in Canada? J Obstet Gynaecol Can 2013 35 434 443 23756274 10.1016/S1701-2163(15)30934-8

[B10] Iniciativa parto normal http://www.federacion-matronas.org/ipn/documentos/iniciativa-parto-normal

[B11] Quintana C Etxeandia I Rico R Armendariz I Férnandez I Grupo de trabajo de la Guía de Práctica Clínica sobre la atención al parto normal Guía dirigida a mujeres embarazadas, a los futuros padres, así como a sus acompañantes y familiares 2010 Vitoria: OSTEBA

[B12] Department of Health Making it better: For mother and baby 2007 London: UK Department of Health

[B13] World Health Organisation Evidence led obstetric care 2005 Geneva: WHO

[B14] Maternity Care Working Party Making normal birth a reality. Consensus statement from the MCWP 2007 London: NCT/RCM/RCOG

[B15] Sociedad Española de Ginecologia y Obstetricia. Protocolo de atención al parto Documentos de consenso 2009 Madrid: SEGO

[B16] Ministry of Health and Consumers’ Affairs Strategy for assistance at normal childbirth in the National Health System 2008 Madrid

[B17] Dirección General de Planificación y Evaluación Plan Estratégico de ordenación de servicios de la atención maternoinfantil en los hospitales de la red pública en Cataluña 2008 Barcelona: Departament de Salut de la Generalitat de Catalunya

[B18] Divisió de Registres de Demanda i d’Activitat Normativa de codificació de les variables clíniques del Registre del Conjunt Mínim Bàsic de Dades dels hospitals d’aguts (CMBD-HA) 2012 Barcelona: Servei Català de la Salut

[B19] Bernal E Aibar C Villaverde MV Abadía MB Martinez N Librero J Peiró S Ridao M Variaciones en la utilización de cesárea en los hospitales públicos del Sistema Nacional de Salud 2010 Zaragoza: Instituto Aragonés de Ciencias de la Salud (I + CS)

[B20] Dahlen H Tracy S Tracy M Bisits A Brown C Thorton C Rates of obstetric intervention among low-risk women giving birth in private and public hospitals in NSW: a population-based descriptive study http://bmjopen.bmj.com/content/2/5/e001723.full 10.1136/bmjopen-2012-001723 PMC3467614 22964120

[B21] Acosta J Xiberta M Rodellar L Jimenez MA Caesarean rates comparison between two groups of doctors at a private hospital in Spain Proceedings of the First European Congress on Intrapartum Care 2013 Amsterdam: European Association of Perinatal Medicine

[B22] Tracy SK Tracy MB Costing the cascade: estimating the cost of increased obstetric intervention in childbirth using population data BJOG 2003 110 717 724 10.1111/j.1471-0528.2003.02045.x 12892682

[B23] Tracy SK Sullivan E Wang YA Black D Tracy M Birth outcomes associated with interventions in labour amongst low risk women: a population-based study Women Birth 2007 20 41 48 10.1016/j.wombi.2007.03.005 17467355

[B24] Eriksen LM Nohr EA Kjaergaard H Mode of delivery after epidural analgesia in a cohort of low-risk nulliparas Birth 2011 38 317 326 10.1111/j.1523-536X.2011.00486.x 22112332

[B25] Moore J Low LK Factors that influence the practice of elective induction of labour: What does the evidence tell us? J Perinat Neonatal Nurs 2012 26 242 250 10.1097/JPN.0b013e31826288a9 22843006 PMC3496748

[B26] Glantz JC Obstetric variation, intervention and outcomes: doing more but accomplishing less Birth 2011 39 286 290 23281946 10.1111/birt.12002

[B27] Pel M Heres M Hart A Van der Veen F Treffers PE Provider-associated factors in obstetric interventions Eur J Obstet Gynecol Reprod Biol 1995 61 129 134 10.1016/0301-2115(95)02129-U 7556833

[B28] Schroeder E Petrou S Patel N Hollowell J Puddicombe D Redshaw M Brocklehurst I Cost effectiveness of alternative planned places of birth in woman at low risk of complications: evidence from Birthplace in England National prospective cohort study BMJ 2012 344 e2292 10.1136/bmj.e2292 22517916 PMC3330132

[B29] McLachlan H Foster D Davey M Lumley J Farrell T Oats J Gold L Waldeström U Alberts L Biro MA COSMOS: Comparing standard maternity care with one-to-one midwifery support: a randomised controlled trial http://www.biomedcentral.com/1471-2393/8/35 10.1186/1471-2393-8-35 PMC2526977 18680606

[B30] EURO-PERISTAconceived, designed and coordinated the study. MP and RE have been involved in acquisition of data and performed statistical analysis. RE and JW drafted the manuscript. HB, CC, IE and XE revised it critically for important intellectual content. JF and VO revised the manuscript and have given the T Project with SCPE and Eurocat. European Health Report The Health and care of pregnant women and babies in Europe in 2010 http://www.europeristat.com

[B31] Cleary-Goldman J Impact of maternal age on obstetric outcome Obstet Gynecol 2005 105 983 990 10.1097/01.AOG.0000158118.75532.51 15863534

[B32] Green JM Baston HA Have women become more willing to accept obstetric interventions and does this relate to mode of birth? Data from prospective study Birth 2007 34 6 13 10.1111/j.1523-536X.2006.00140.x 17324172

[B33] Bell JS Campbell DM Graham WJ Gillian CP Ryan M Hall MH Do obstetric complications explain high caesarean section rates among women over 30? A retrospective analysis BMJ 2001 322 894 895 10.1136/bmj.322.7291.894 11302901 PMC30584

[B34] McPherson K Gon G Scott M Organisation for Economic Co-Operation and Development International variations in a selected number of surgical procedures OECDE Health working papers 2013 Paris Health working Paper. N 61

[B35] VPM Grupo Atlas Validación de indicadores de calidad utilizados en el contexto internacional: Indicadores de seguridad de pacientes e indicadores de hospitalización evitable 2008 Madrid: Ministerio de Sanidad y Consumo

[B36] Redondo A Sáez M Oliva P Soler M Arias A Variabilidad en el porcentaje de cesáreas y en los motivos para realizarlas en los hospitales españoles Gac Sanit 2013 27 258 262 10.1016/j.gaceta.2012.08.001 23246252

[B37] Davis D Baddock S Pairman S Hunter M Benn C Wilson D Dixon L Herbirson P Planned place of birth in New Zealand: Does it affect mode of birth and intervention rates among low-risk women? Birth 2011 38 111 119 10.1111/j.1523-536X.2010.00458.x 21599733

[B38] Goldvall K Waldeström U Tingstig C Gruneland C In-Hospital Birth Center with the same medical guidelines as standard care: a comparative study of obstetric interventions and outcomes Birth 2011 38 120 128 10.1111/j.1523-536X.2010.00461.x 21599734

[B39] Tracy SK Sullivan E Dahlen H Black D Wang YA Tracy MB General obstetrics:Does size matter? A population-based study of birth in lower volumen maternity hospitals for low risk women BJOG 2006 113 86 89 16398776 10.1111/j.1471-0528.2005.00794.x

[B40] Maceira MC Salgado A Atienza G La asistencia al parto de las mujeres sanas. Estudio de la variabilidad y revisión sistemática 2009 Madrid: Ministerio de Ciencia e Innovación

[B41] Roberts CL Bell JC Ford JB Morris JM Monitoring the quality of maternity care: how well are labour and delivery events reported in population health data? Paediatr Perinat Epidemiol 2009 23 144 152 10.1111/j.1365-3016.2008.00980.x 19159400

[B42] Knight HE Gurol I Mahmood TA Templeton A Richmond D Van der Meulen JH Cronwell DA Evaluating maternity care using national administrative health databases: How are statistics affected by the quality of data on method of delivery http://www.biomedcentral.com/1472-6963/13/200 10.1186/1472-6963-13-200 PMC3673850 23721128

